# Superior clavicle plate with lateral extension for displaced lateral clavicle fractures: a prospective study

**DOI:** 10.1007/s10195-013-0228-0

**Published:** 2013-02-12

**Authors:** Davut Tiren, Joseph P. A. M. Vroemen

**Affiliations:** Department of Surgery, Amphia Ziekenhuis, Breda, The Netherlands

**Keywords:** Displaced lateral clavicle fracture, Distal clavicular fracture, Superior clavicle plate with lateral extension, Locked clavicle plate

## Abstract

**Background:**

Until now there have been no prospective studies describing the results of using the superior clavicle plate with lateral extension in patients with displaced lateral clavicle fractures (Neer type 2). The purpose of this study was to evaluate the results of applying this plate for this specific type of fracture.

**Materials and methods:**

In this prospective study, seven patients (mean age 43, M:F; 6:1) with a fresh displaced lateral clavicle fracture were evaluated with a mean follow-up of 10 months. Analysis included functional and subjective outcome, time until union, time until return to work, and complications.

**Results:**

All patients achieved clinical and radiological union within 6–12 weeks. Full range of motion as well as a return to work was achieved in most cases within 2 weeks. The mean Constant score was 98 (range 90–100), the DASH score was 3.6 (range 0–11.4), and the Shoulder Rating Questionnaire score was 97 (range 96–100). No major complications were encountered. Three patients required plate removal: two because of a prominent and subcutaneous plate and one because of an intra-articular screw.

**Conclusions:**

In this study, use of the superior clavicle plate with lateral extension yielded excellent results in the treatment of this difficult fracture. In particular, patients acquired full range of motion within 2 weeks, reflecting the stability of the osteosynthesis achieved with this implant.

## Introduction

The treatment of displaced lateral clavicle fractures is controversial. Conservative treatment may lead to good functional results in a selected group of patients. Even though nonunion occurs in this group, it can be asymptomatic [1, 2]. However, in a substantial group of patients, especially the young and active, functional impairment and pain after this fracture can lead to invalidity [1, 2]. Operative stabilization leads to a high percentage of union with good functional results. Since Neer [3, 4] first described fixing these fractures with two transarticular K-wires, many techniques and methods have been described for fixation. This indirectly suggests that these methods do not always yield the desired results [5]. In the last decade, the hook plate—originally developed by Balser to treat acromioclavicular dislocations—has been used as treatment for this difficult fracture [6]. However, because of impingement complaints due to the close relationship to the rotator cuff and the acromioclavicular joint (ACJ), as well as the obligation to remove the implant after fracture union, this plate has gained some negative publicity.

All of the different methods that are employed to fix these fractures have been proposed because of the difficult nature of these fractures. Due to the soft, short distal metaphyseal end of these types of fractures, it is impossible to fix this part of the bone with conventional plates and screws with sufficient stability to allow early active mobilization of the shoulder.

Kalamaras [7], Daglar [8], and Herrmann [9] described series of 9, 14, and 7 patients, respectively, with a displaced lateral clavicle fracture. The implant used in these studies was a volar distal radius plate with locked screws. They demonstrated that it was possible to sufficiently fix this difficult fracture due to new developments in the plate–screw interface.

In 2010, the LCP superior clavicle plate with lateral extension became available to our department. This plate allows fixation of the lateral end of the fracture with six 2.7 mm locked screws that diverge. This ensures good screw purchase in the soft metaphyseal bone and increases the pull out strength without interfering with the ACJ.

Until now, there have been no prospective studies that describe the results of using the superior clavicle plate with lateral extension. We evaluated the results of applying this plate in our patient population by setting up a study in our hospital, as described below.

## Materials and methods

### Patients

All consecutive patients in 2011 with a displaced lateral clavicle fracture who were operated on in our hospital within 4 weeks after the injury using the superior clavicle plate with lateral extension were included in this study and followed prospectively. Analysis included functional and subjective outcome, time until union, time until return to work, and complications.

### The implant

**Fig. 1:**
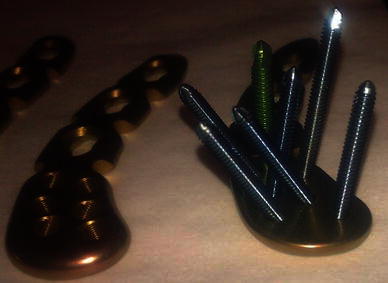
Clavicle plate with lateral extension: top–bottom view

**Fig. 2:**
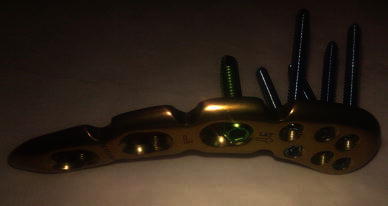
Clavicle plate with lateral extension—side view

The Synthes^©^ LCP superior clavicle plate with lateral extension (Figs. 1, 2) is a precontoured locking compression plate with a medial part that accepts 3.5 locking or cortical screws and a lateral end that accepts 2.4 cortical or 2.7 locking screws. The lateral end measures 2 cm and is wider, adapting to the shape of the lateral end of the clavicle. On the lateral end it contains six screw holes that diverge for increased pull-out strength. There are right and left versions that allow 3–8 holes on the medial part.

### Surgical technique

All operations were performed by two trauma surgeons. The patients were operated on in the beach chair position under general anesthesia with the arm on the affected side, freely movable. Either an inline incision parallel to the clavicle or a sabre-cut incision medial to the fracture side was used. Full-thickness skin flaps were developed. The fracture was reduced; large comminuted fragments were temporarily fixed with K-wires. Repair of torn ligaments was not performed. Interposed tissue was removed. Without opening the ACJ, the location of the joint was marked with a needle and confirmed with fluoroscopy. The lateral end of the plate was centered in the anteroposterior direction and with the most lateral end of the plate parallel to the ACJ. If necessary, bending or twisting of the plate was performed to adjust to the individual anatomy. The plate was provisionally fixed with a 3.5 mm cortical screw on the shaft, and the lateral end was fixed with the locked 2.7 mm screws. Finally, the other holes in the plate were filled with locked 3.5 mm screws. The full-thickness layers were closed over the plate, after which the skin was closed.

### Follow-up

All patients started active movement of the extremity directly postoperatively under the supervision of a physiotherapist. If necessary, out-patient physiotherapy was continued. Shoulder movement above 90° was not limited, but heavy labor and contact sports were discouraged. All patients were discharged the day after operation.

Patients were followed up according to a standard protocol in which they were clinically and radiographically assessed at 2, 6, 12, 18, and 24 weeks. At 24 weeks, objective and subjective shoulder function was measured using three scoring systems: the Constant scoring system, the QuickDASH, and the Shoulder Rating Questionnaire.

### Patient reported outcome and clinical assessment

The Constant score [10] consists of four individual parameters which contribute a maximum of 100 points in total: pain (15 points), activities of daily living (20 points), range of motion (40 points), and strength (25 points). The score was also compared with the contralateral shoulder. These scores were graded as excellent (90–100 points), good (80–89 points), fair (70–79 points), or poor (<70 points).

The Shoulder Rating Questionnaire (SRQ) is a questionnaire that assesses shoulder symptoms and function in addition to the level of social participation, with a possible range of 17–100 points [11]. A Cochrane review validated the Shoulder Rating Questionnaire [12].

The QuickDASH [13] is a shortened version of the DASH outcome measure that uses 11 items to measure physical function and symptoms in people with any musculoskeletal disorders of the upper limb. It is valid, reliable and responsive when used for research or clinical purposes. It has a possible range of 0–100, with 0 being the best score and 100 the worst.

### Statistics

No statistical analysis was performed.

## Results

### Demographics

Seven patients with a displaced lateral clavicle fracture (Neer type 2) were treated by two trauma surgeons (Table 1). Six of these patients were male and one was female, with a mean age of 43 years (24–60). All fractures but one occurred due to a fall on the arm during outdoor sporting activities like bicycling, jogging, and horse riding. One patient with a contralateral midshaft clavicle fracture was involved in a car accident (patient 5).

**Table 1:** Patients, results, and complications

Nr	Age	Sex	Side/dominance	Profession	Trauma mechanism	Incision	Plate and screws used	Time to union on X-ray (weeks)	Return to work	Functional ROM	Plate removed	Total follow-up (months)	Constant/% of other side	Quick DASH	SRQ	Complication
1	49	M	L/R	Technical advisor	Bike racing	Sabre-cut	4-hole plate, 4 shaft screws, 6 lateral screws	6	3 days	2 weeks	No	12	100/100	2.3	100	
2	34	M	L/L	Process operator	Jogging	In-line	4-hole plate, 4 shaft screws, 4 lateral screws	6	6 weeks	2 weeks	Yes, 9 months	11	100/100	2.3	96	Subcutaneous plate 1 screw in ACJ
3	46	M	L/R	Production leader	Horseback riding	In-line	4-hole plate, 3 shaft screws, 6 lateral screws	12	5 days	2 weeks	Yes, 4 months	11	98/98	4.5	96	Lateral screw in ACJ
4	60	M	L/R	Sports instructor	Bike racing	Sabre-cut	3-hole plate, 3 shaft screws, 4 lateral screws	6	1 week	2 weeks	No	10	100/100	0	100	
5	24	F	L/R	Student	Car accident	Sabre-cut	3-hole plate, 2 shaft screws, 4 lateral screws	12	1 week	6 weeks	No	10	90/100	11.4	89	
6	42	M	L/R	Small business owner	Bike racing	Sabre-cut	4-hole plate, 3 shaft screws, 6 lateral screws	6	3 days	2 weeks	No	8	100/100	0	100	
7	45	M	R/R	Service station attendant	Bike racing	In-line	3-hole plate, 2 shaft screws, 4 lateral screws	12	4 weeks	6 weeks	Yes, 5 months	6	100/100	4.5	96	Subcutaneous plate

*M* male, *F* female, *L* left, *R* right, *ROM* range of movement, *SRQ* Shoulder Rating Questionnaire, *ACJ* acromioclavicular joint

Six left-sided fractures were operated on, and two patients had an operation on the dominant side.

**Fig. 3:**
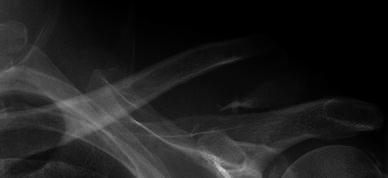
A displaced lateral clavicle fracture

**Fig. 4:**
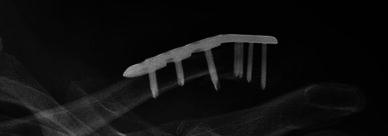
After reduction and fixation

**Fig. 5:**
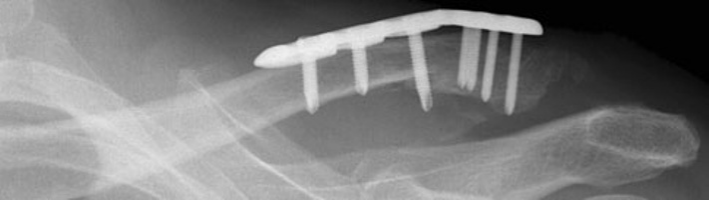
After complete healing of the fracture

A four-hole plate was used in four cases and a three-hole plate in three cases. Only four of the six lateral holes were filled with 2.7 mm screws in four cases, and all six lateral holes were filled in three cases (Figs. 3, 4, 5).

### Postoperative course

Five of the seven patients had a functional range of movement of the shoulder during the first outpatient visit at 2 weeks. All patients had a functional range of movement at 6 weeks. All patients had returned to normal daily activities and to work without limitations at 6 weeks.

All fractures had united without further surgical intervention at 12 weeks, with a mean time to radiological union of 8 weeks (Table 1).

### Complications

Major complications such as infection, implant failure, shoulder instability, and rotator cuff damage were not observed, although some minor complications did occur. Patients 2 and 7 experienced some discomfort due to the subcutaneous and prominent position of the plate, even though no wound-healing problems occurred. Patient 3 developed pain with limited functional impairment due to the intra-articular position of the most lateral anterior 2.7 mm screw. Initially this patient had no complaints. At 12 weeks he mentioned some discomfort with strenuous activities, and at 18 weeks he had a functional relapse due to pain. After plate removal at 24 weeks’ follow-up, he regained optimal function with a slight limitation in shoulder function which translated to a Constant score of 98. This complication of a perforating screw—the most lateral posterior one—in the ACJ was also seen in patient 2.

These three patients required plate removal at 4, 5, and 9 months.

### Patient-reported outcome scores

The functional outcome of each patient, as calculated via the Constant score (with a mean of 98), the QuickDASH score (with a mean of 3.6), and the subjective outcome as evaluated by the SRQ (with a mean of 96.7), is shown in Table 1. The functional outcome compared to the contralateral side was graded excellent in all patients.

## Discussion

Many different operative techniques for treating the displaced lateral clavicle fracture have produced satisfactory functional results, but all have known drawbacks and complications due to the nature of the displaced lateral clavicle fracture. The fracture unites when the forces distracting the fracture ends are neutralized. The latter can be a challenge because of the small, soft, usually comminuted distal metaphyseal fragment and the proximity to the AC joint. Many techniques and methods for achieving reduction and fixation have been described. These can be divided into the following categories:

- Reducing the fracture ends and transacromially fixing with K-wires with or without a wire cerclage
- Indirectly reducing the fracture ends by fixing the medial clavicle to the coracoid process using either a screw (Bosworth), nonresorbable slings, or ropes
- Reducing the fracture ends medially using a classical plate and screw interface, and on the lateral side with a hook that is positioned under the acromion (clavicle hook plate)
- By reducing and fixing the fracture ends with a plate and (locked) screws at both ends (T-shaped distal radius plates, clavicle plates with lateral extension)

Each operative method has its own specific drawbacks and complications. K-wires with cerclage have a high rate of infection and nonunion due to migration of the K-wires [5].

Indirect reduction with a screw, slings, or ropes requires extensive dissection to expose the fracture and the coracoid process. Erosion and fractures due to bore holes that have to be made through both structures is a known complication [14]. Another issue with this technique is limitation on the rotation of the clavicle due to the coracoclavicular fixation, which prolongs the time to full recovery [15].

In the last decade, the clavicle hook plate has been widely utilized for this type of fracture, and has been found to yield good results, particularly because the rigidity of the fixation makes early postoperative motion possible. Even though mid-term results of treatment of this plate indicated that there were no adverse effects on the ACJ, it is associated with several uncomfortable short-term complications—such as subacromial impingement and subacromial osteolysis accompanied by pain—in a substantial proportion of the patients treated using this plate, thus requiring plate removal [16].

A stable plate osteosynthesis is achievable with locking plates in these fractures, as described by Kalamaras, Daglar, and Herrmann. Although the results achieved by these authors with distal radius plates are promising, they used plates that were not intended for this fracture. The single distal locking row in the distal radius plates used in these studies are meant to buttress the articular surface of the distal radius in a raft fashion, with all the screws pointing in the same direction. The most significant complication described by these studies was pull-out of the plate when the plate had too little grip in a small or osteoporotic lateral fragment. Some authors advise suture augmentation of the coracoclavicular ligaments or coracoclavicular fixation in such cases [7, 9]. In our opinion, pull-out occurred in these cases for these plates because they did not neutralize the downward forces acting on the lateral end of the fracture sufficiently.

The specifically designed superior clavicle plate with lateral extension evaluated in this study utilizes a better construct for the lateral fragment: three rows with double screws that have diverging screw angles. This ensures much more stability against the pull-out and shear forces that act on the short and soft metaphyseal fragment. We did not encounter pull-out of the plate in our series, even in cases where both coracoclavicular fragments were avulsed from the main fragments.

A complication we encountered with this plate in our series was perforation of the AC joint by the most lateral screws. The angles of the screws are predetermined by the plate. The individual anatomy of the lateral end of the clavicle must be taken into consideration as well as the angle of the screws when positioning the plate. Patients with perforation of the AC joint required plate removal to relieve symptoms.

Another minor complication we encountered was the prominent position of the plate. Since the plate is precontoured, there is seldom reason to bend the plate, but when the plate does need to be bent to adjust it to the individual shape of the clavicle, we think a prominent and palpable plate would be much less of a problem.

In this study, most patients regained pre-injury levels of activity within a very short period after the operation. In our opinion, this was due to the stability of the construct, which adequately immobilized the fracture and allowed early pain-free postoperative mobilization. The high Constant score and the low QuickDASH score in combination with the high SRQ indicate normal shoulder function with little or no sign of any negative influence on daily activities.

We regard the LCP superior clavicle plate with lateral extension as a good implant for fixation of the displaced lateral clavicle fracture. The individual anatomy at the level of the lateral clavicle and the close relation to the ACJ requires profound knowledge of the anatomy. The procedure itself requires adequate surgical exposure, anatomic reduction, and experience with this specific plate.

The main limitation of this series is the very small number of patients included in the study. Analyzing a small number of patients can introduce several biases, so any conclusions drawn should be interpreted accordingly, with caution. However, the rarity of this type of fracture makes it difficult to recruit many patients with this type of fracture for a study.

In this prospective study, we obtained excellent initial results with the superior clavicle plate with lateral extension in our patients, although the number of patients included in the study was small. This implant is, in our opinion, the most sophisticated of the many techniques and implants that have been applied to treat such fractures. It does not violate the surrounding structures when used correctly, and it fixes the fracture sufficiently to provide a rigid and stable osteosynthesis, with the possibility of early postoperative mobilization and a short time to union.
